# Is AI 3D-printed PSI an accurate option for patients with developmental dysplasia of the hip undergoing THA?

**DOI:** 10.1186/s12891-024-07449-3

**Published:** 2024-04-22

**Authors:** Han Zheng, Eryou Feng, Yao Xiao, Xingyu Liu, Tianyu Lai, Zhibiao Xu, Jingqiao Chen, Shiwei Xie, Feitai Lin, Yiling Zhang

**Affiliations:** 1 Department of Arthrosis Surgery, Fuzhou Second Hospital, Fuzhou, China; 2 Fujian Provincial Clinical Medical Research Center for First Aid and Rehabilitation in Orthopaedic Trauma(2020Y2014), Fuzhou, China; 3 The Second School of Medicine, Fujian University of Traditional Chinese, Fujian, China; 4 Fuzhou Changle Hospital of Traditional Chinese Medicine, Fuzhou, China; 5 Longwood Valley Medical Technology Co Ltd, Beijing, China

**Keywords:** Artificially Intelligent, 3D-Print, PSI, THA, DDH

## Abstract

**Background:**

In traditional surgical procedures, significant discrepancies are often observed between the pre-planned templated implant sizes and the actual sizes used, particularly in patients with congenital hip dysplasia. These discrepancies arise not only in preoperative planning but also in the precision of implant placement, especially concerning the acetabular component. Our study aims to enhance the accuracy of implant placement during Total Hip Arthroplasty (THA) by integrating AI-enhanced preoperative planning with Patient-Specific Instrumentation (PSI). We also seek to assess the accuracy and clinical outcomes of the AI-PSI (AIPSI) group in comparison to a manual control group.

**Methods:**

This study included 60 patients diagnosed with congenital hip dysplasia, randomly assigned to either the AIPSI or manual group, with 30 patients in each. No significant demographic differences between were noted the two groups. A direct anterior surgical approach was employed. Postoperative assessments included X-rays and CT scans to measure parameters such as the acetabular cup anteversion angle, acetabular cup inclination angle, femoral stem anteversion angle, femoral offset, and leg length discrepancy. Functional scores were recorded at 3 days, 1 week, 4 weeks, and 12 weeks post-surgery. Data analysis was conducted using SPSS version 22.0, with the significance level was set at α = 0.05.

**Results and conclusion:**

The AIPSI group demonstrated greater prosthesis placement accuracy. With the aid of PSI, AI-planned THA surgery provides surgeons with enhanced precision in prosthesis positioning. This approach potentially offers greater insights and guidelines for managing more complex anatomical variations or cases.

## Background

Patients with Developmental dysplasia of the hip (DDH) undergoing total hip arthroplasty (THA) encounter three primary challenges: acetabular reconstruction, soft tissue balancing around the hip joint, and femoral canal preparation. Notably, on the acetabular side, patients classified as Crowe III or IV often exhibit inadequate coverage of the femoral head by the acetabulum, alongside a diminutive and shallow acetabulum with defects in the anterior and lateral walls. These conditions complicate the identification of the true acetabulum during surgery, potentially leading to incorrect placement of the acetabular prosthesis. Such misplacement may result in prosthesis loosening due to insufficient support [1].

The direct anterior approach (DAA) for THA is characterized by a steep learning curve and demands extensive skill in preoperative planning and intraoperative techniques from surgeons. As for surgical navigation, only a limited number of hospitals are equipped with robotic systems, leaving the majority of hip arthroplasties to depend on the surgeon's expertise and approximate measurements [2]. While seasoned surgeons are able to achieve satisfactory surgical outcomes using these methods, less experienced practitioners may struggle to obtain optimal results, potentially increasing the risk of postoperative complications [3].

According to reports, the incorporation of technologies such as robotics, navigation, and fluoroscopic-based systems has enhanced the precision of implant placement in THA. The benefits of PSI include its user-friendliness and the facilitation of more accurate outcomes [4]. Further, advancements in artificial intelligence and 3D printing technologies have shown promising potential in medical applications, notably in enhancing the efficiency of PSI production.

Therefore, this clinical study aims to develop an artificial intelligence-enhanced, 3D printed PSI to facilitate accurate placement of hip socket and femur implants via a direct anterior approach. It seeks to reduce surgical duration and assess whether PSI can yield superior clinical outcomes and implant placement precision.

## Methods

Our facility received approval from the institutional review board. Between November 1, 2021, and May 31, 2022, we collected data from 60 patients undergoing THA treatment. Included patients met specific criteria: they had not undergone previous hip replacement surgery on either side; had not been subjected to shelf procedures, femoral osteotomies, or other hip surgeries; underwent 3D preoperative planning based on computerized tomography (CT) at our institution; received elective primary THA; were diagnosed with DDH; and provided written informed consent. Exclusion criteria were refusal to participate, the presence of a metal prosthesis or infection in the same hip joint. Patients were randomized based on bed number, with nurses managing care blinded to the study groups. Thirty patients received PSI-assisted THA, and thirty underwent conventional THA. No statistically significant differences were found between the two groups in terms of age, gender, height, weight, Body Mass Index (BMI), among other factors (Table 1). There were no instances of complications such as dislocation, periarticular infection, intraoperative fracture, or periprosthetic fracture in either group. Preoperative hemoglobin (HGB) levels showed no significant difference between the groups (*P* > 0.05).

**Table 1:** Demographic characteristics

	AIPSI group		Control group		*P* Value
	(*n* = 30)		(*n* = 30)		
Age in years	64.3 ± 10.0		61.3 ± 10.2		0.256
BMI(kg/m^2^)	24.4 ± 2.4		23.8 ± 2.5		0.340
Hemoglobin(g/L)	130.9 ± 17.8		131.3 ± 15.3		0.932
Crowe type*	I = 15	II = 9	I = 9	II = 13	0.436
	III = 3	IV = 3	III = 5	IV = 3	
Sex*	Men = 7 Women = 23		Men = 9 Women = 21		0.559

BMI body mass index

Presented as the mean ± standard deviations, except for those with* which are presented as n

### Preoperative planning

Patients in both the PSI and control groups underwent CT-based 3D preoperative planning using AIHIP software (Version 3.0, Longwood Valley Technology, China), facilitated by two orthopedic surgeons. In contrast to other image processing software, which often involves numerous parameters and complex usability [5], AIHIP was designed to automate preoperative evaluations and simulate postoperative results. Convolutional neural networks (CNNs) were trained to automatically segment images, distinguishing between the femur and pelvis, and to identify key anatomical landmarks. The software then adjusted the pelvic orientation to a neutral stance using the bilateral anterior superior iliac spines and the pubic symphysis plane as references.

Before surgery, a detailed dysmorphic evaluation using established biomarkers was conducted. This evaluation first determined the position and size of the acetabular prosthesis, making adjustments to the acetabular cup's abduction and anteversion angles, as well as its coverage. The process then specified the position and size of the femoral prosthesis and automatically established the level of femoral resection. Simulations to forecast post-operative outcomes were performed, illustrating potential differences in leg length and modifications in femoral offset.

### Patient-specific instrument

**Fig. 1:**
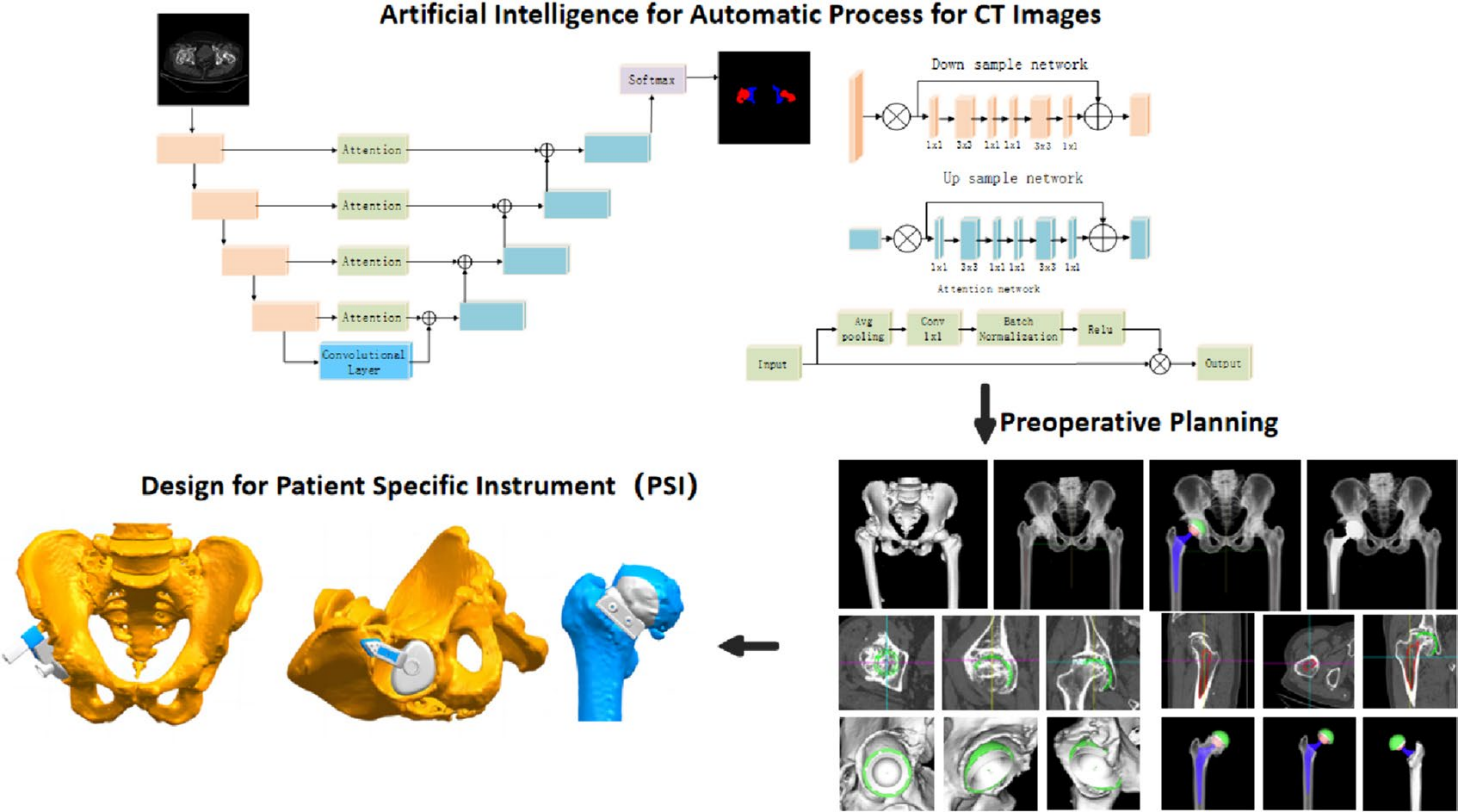
The design process for Patient-Specific Instruments (PSI) employs artificial intelligence for the automated processing of computed tomography (CT) scan images. This preoperative planning, grounded in the processed CT images, facilitates the determination of the PSI's size and position

The PSI's design was preoperatively planned as illustrated in Fig. 1, using a three-dimensional planning system to simulate the PSI’s size, position, and orientation. The objective was to maximize the contact area while reducing surgical exposure. The design for each PSI received validation from two orthopedic surgeons, followed by the production of 3D models and surgical templates for the acetabulum and proximal femur according to the preoperative plan. The PSI comprised two components: one for guiding horizontal and directional cuts of the proximal femur, and another for directing the acetabulum's cup reaming and implant placement. The average duration from CT data acquisition to PSI completion was under 12 h, including CT processing within 1 h, THA planning and PSI simulation within 1 h, and 3D printing within 8 h.

### Radiographic outcomes

Postoperative evaluation for each case involved assessing anterior–posterior X-rays of the pelvis, executed by a skilled technician with more than 10 years of experience in musculoskeletal imaging. This technician adjusted the tilt, rotation, and magnification to secure standard hip joint views. All postoperative imaging results were anonymized. Measurements were carried out by an experienced orthopedic resident with over 4 years of clinical experience. Before the study commenced, the resident underwent training in standardized radiographic measurements to ensure accuracy and consistency. The average of the resident's two measurements was utilized for statistical analysis. Follow-up X-ray examinations were conducted on the patients at least 4 weeks post-surgery.

The Lewinnek method [6, 7] was employed to measure the acetabular abduction angle and the acetabular anteversion angle. The abduction angle is defined by the angle between the line connecting the bilateral ischial tuberosities and the line through the center of the acetabular cup. The formula for the acetabular anteversion angle is given by: acetabular anteversion angle = ARC sine(a/b). The safe zone for implant placement, as defined by Meermans and Abdel et al. [8–10] and supported by previous research, is an abduction angle of 40 ± 10° and an anteversion angle of 15 ± 10°. Leg length discrepancy (LLD) was identified as the variance in vertical distance from the inferior margin of the lesser trochanter to the bilateral teardrop lines. The difference in femoral offset (FO) was calculated as the discrepancy in vertical distance between the centers of the two femoral head prostheses and the proximal center axis of the femur [11].

### Functional outcomes

Functional outcomes were assessed using the Harris hip score and the visual analog scale (VAS) for pain score preoperatively, at 3 days, and at 1, 3, and 12 months postoperatively.

### Other outcomes

Blood loss data were extracted from surgical records. The number of bone cuts was determined by the frequency of oscillating saw use for femur segmentation. The number of acetabular reaming iterations performed before finalizing implant selection was recorded. Hemoglobin (HGB) levels were measured preoperatively (7 days before surgery) and 3 days postoperatively. A decrease in HGB levels was identified as the difference between preoperative and postoperative measurements. The agreement rate between planned and actual implant placement was calculated, considering a variance of one implant model as acceptable.

### Surgical technique and perioperative management

**Fig. 2:**
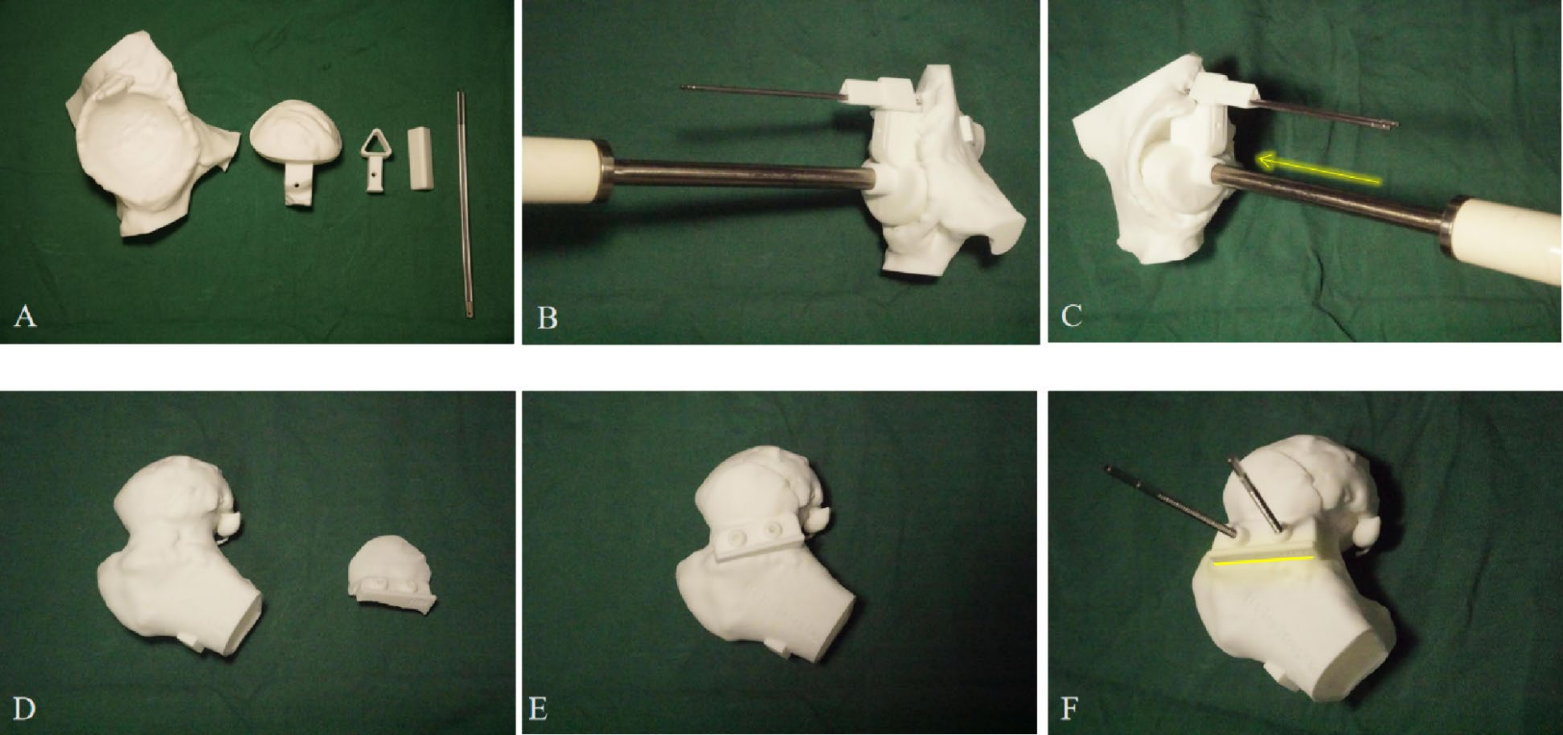
Utilization of 3 dimensional-printed PSI, **A**. Acetabular parts of PSI; **B**. Insertion of the fitting guide to locate the designed position and direction, with additional parts assembled following the Lego principle, and two pins secured through designated holes; **C**. Removal of the fitting guide, with the reaming direction aligned parallel to the pins, as indicated by the yellow arrow; **D**. Femoral components of PSI; **E**, **F**. The resection guide was anchored with 2 pins, and both the level and direction of resection were established by the resection guide, denoted by the yellow line

**Fig. 3:**
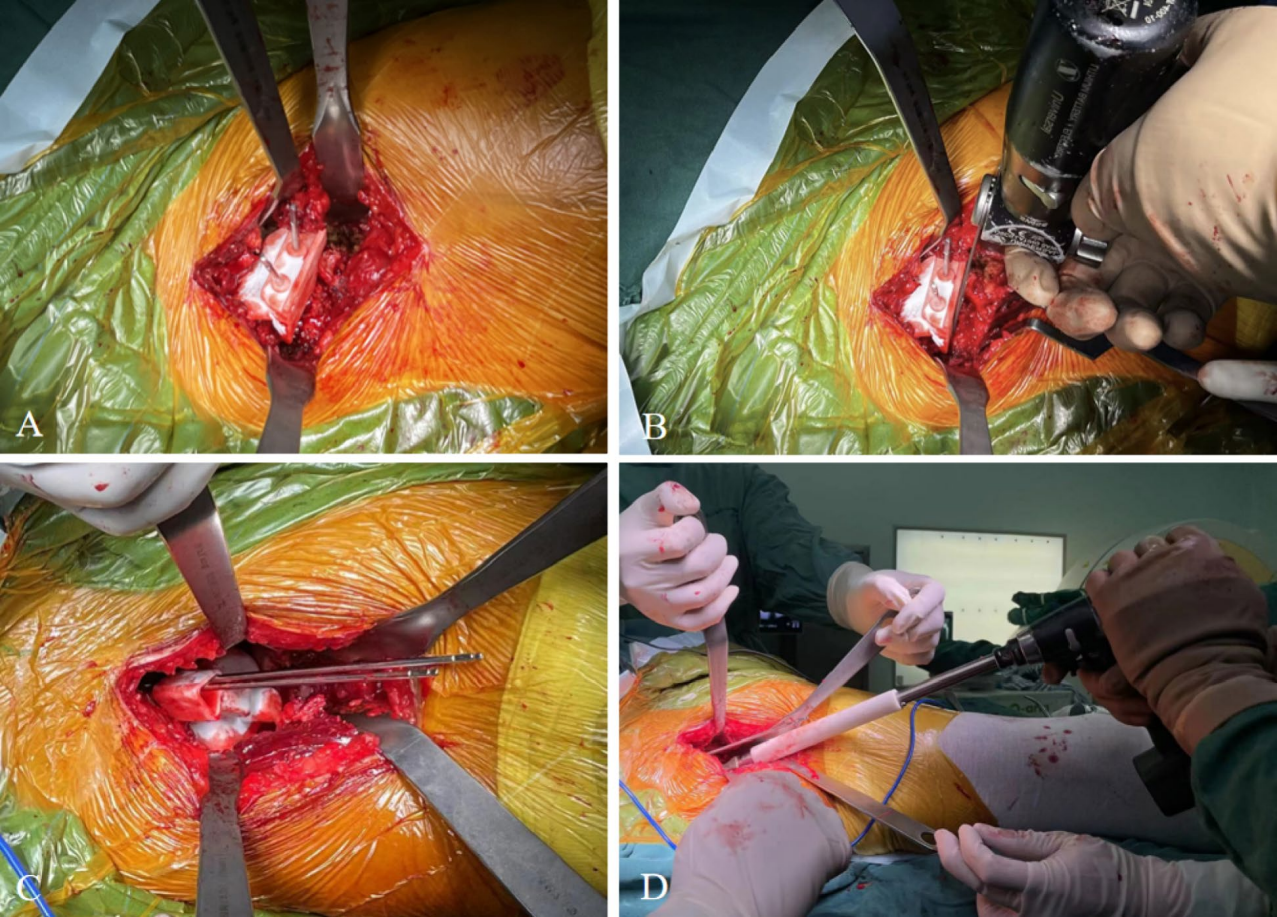
Intraoperative application of 3-dimentional printed PSI: **A**. The resection guide was anchored with 2 pins; **B**. The resection guide determined the level and direction of resection; **C**. The two anchored pins served as guiding pins, positioned in parallel; **D**. The angle between the guiding pins and the body axis defined the direction of the acetabular reaming abduction angle

All THA procedures were conducted by an experienced surgical team employing a uniform anterior approach technique, which was novel to the team in the context of anterior THA PSI guide usage. The standard direct anterior approach was adopted. Technical specifics included positioning patient’s supine under general anesthesia, with standard preparation and draping of the surgical site. Using the direct anterior route, the femoral neck was sequentially exposed. In the PSI group, the cutting guide was positioned between the femoral neck and anterior bony structures as determined by preoperative planning, guiding the femoral neck cut. Following femoral head removal and acetabular exposure, the superior joint capsule and labrum were excised, and the bone was delicately detached. The round ligament was severed, and the obturator foramen identified. Prosthetic design matched the femoral head and original acetabulum sizes. Reaming guide placement and assembly followed the "Lego principle," which involves completing the assembly and utilization of the guide structure within the patient's incision.. Guide pins, fixed in parallel, determined the anterior tilt angle for acetabular reaming based on their angle with the horizontal plane and the abduction angle from their orientation to the body axis. PSI application is depicted in Fig. 2 and 3. Subsequently, femoral preparation included proximal opening and medullary cavity broaching, with femoral implant anterior tilt angle aligned to the distal femoral canal line direction. The control group similarly prepared the acetabulum, maintaining a 40° abduction angle and a 15° anterior tilt angle during reaming, before implant insertion. Post-implantation, limb length was measured, and hip stability assessed, followed by layered incision closure. C-arm fluoroscopy verified implant positioning for both groups. The protheses used were Pinnacle Cup (DePuy Orthopaedics, Warsaw, IN), Corail Stem (DePuy Orthopaedics), routine procedure involved screws, excluding femoral head allografts, augments, or cement. The lead surgeon documented bone cuts and reaming instances. Standard perioperative care and patient education were uniformly provided to all patients.

### Data analyses

Data analysis was conducted using SPSS version 22.0. Continuous data were represented as mean ± standard deviation. The independent t-test was used for comparisons between groups, while the paired t-test facilitated pre- and post-operative comparisons within groups. For categorical data, rates were provided, with the chi-square test or Fisher's exact test applied to intergroup comparisons. A significance level of α = 0.05 was established.

## Results

**Fig.4:**
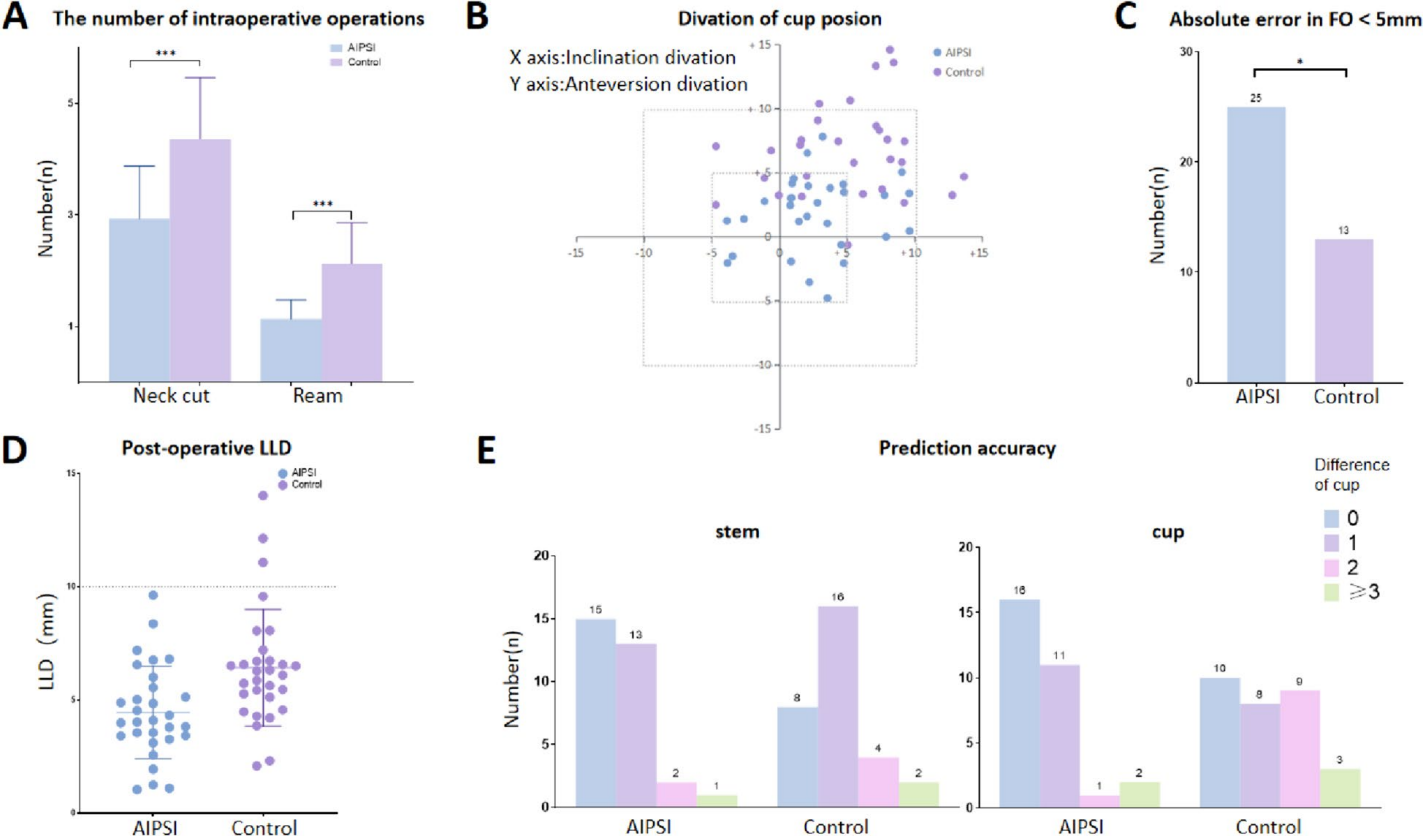
Postoperative outcomes. **A** Number of intraoperative neck cuts and reams; **B**. Discrepancy between preoperative and postoperative cup inclination and anteversion; **C**. Postoperative femoral offset discrepancy; **D**. Postoperative leg length discrepancy; **E**. Accuracy in predicting cup and stem placement for AIPSI and Control methods

The AIPSI group showed a significant reduction in both neck cuts and reams. Specifically, the mean absolute error for neck cut frequency in the AIPSI group was 1.1 ± 0.4 mm, with 87% (26/30) of cases requiring only one cut, compared to the Control group at 2.1 ± 0.7 mm (*P* < 0.001) and 20% (6/30) necessitating just one cut. The AIPSI group had an average of 2.9 ± 0.9 reams, versus the Control group's 4.4 ± 1.1 reams (*P* < 0.001) (Fig. 4A).

In terms of achieving the target inclination angle within a 5° margin of error, the Control group saw an increase in the number of cases failing to meet this criterion, with 17 cases, as opposed to only 5 in the PSI group (*P* = 0.003). For a 10° margin of error, no cases in the PSI group failed, compared to 2 in the control group (*P* = 0.492). The PSI group also had significantly fewer cases failing to achieve the target anteversion angle within a 5° margin of error, with only 3 cases, against 19 in the Control group (*P* < 0.001). Within a 10° margin, no cases in the PSI group failed, whereas there were 5 in the Control group (*P* = 0.052) (Fig. 4B).

The mean absolute error for cup inclination was 2.9 ± 1.8°in the PSI group versus 7.4 ± 4.3°in the free-hand group (*P* < 0.001). For cup anteversion, the mean absolute error was 3.7 ± 2.7°in the PSI group versus 5.6 ± 3.5°in the free-hand group (*P* = 0.002) (Fig. 4B).

The AIPSI group exhibited significantly better performance in achieving the desired femoral offset (FO), with 83.3% (25/30) achieving an offset within a ± 5 mm margin of error, compared to only 56.7% (13/30) in the control group (*P* = 0.047) (Fig. 4C).

The mean postoperative LLD was 4.5 ± 2.0 mm in the PSI group versus 6.4 ± 2.9 mm in the free-hand group (*P* < 0.001)( Fig. 4D). LLD exceeded 10 mm in three cases within the control group, while no cases in the PSI group experienced LLD beyond this threshold (*P* = 0.052)(Table 2).

**Table 2:** Absolute error between preoperative planning and postoperative outcomes

	AIPSI Group	Control Group	*P* Value
Acetabular Component			
Inclination Error > 5( °) *	5(16.7)	17(46.7)	0.003
Inclination Error > 10( °) *	0(0)	2(6.7)	0.492
Anteversion Error > 5°( °) *	3(10.0)	19(63.3)	<.001
Anteversion Error > 10°( °) *	0(0)	5(16.7)	0.052
Femoral Component			
Normal FO (< ± 5 mm compared to CL) *	25(83.3)	13(43.3)	0.047
Postoperative Leg Length Discrepancy (LLD)			
LLD (mm)	4.5 ± 2.0	6.4 ± 2.9	0.004
LLD > 10*	0(0)	3(10.0)	0.236

*LLD* leg length discrepancy, *FO* Femoral offset *CL* compared to the contralateral normal hip

Presented as the mean (ranges), except for those with* which are presented as n (%)

### Operation time and blood loss

The average operation duration was 79.7 ± 20.8 min for the AIPSI group compared to 87.0 ± 26.6 min for the Control group (*P* = 0.235). The mean blood loss amounted to 405.7 ± 176.6 mL in the PSI group and 397.0 ± 188.3 mL in the control group (*P* = 0.855) (Table 3).

**Table 3:** Operation time and blood loss

	AIPSI group	Control Group	*P* Value
Operating Time(min)	79.7 ± 20.7	87.0 ± 26.7	0.235
Blood Loss(mL)	405.7 ± 176.6	397 ± 188.3	0.855

Presented as the mean ± standard deviations

### Functional outcome

To assess postoperative functional outcomes, the Harris score and the Visual Analog Scale (VAS) pain score were evaluated preoperatively, 3 days postoperatively, and at 1, 3, and 12 months postoperatively (Table 4). There were no significant differences in preoperative Harris scores or VAS pain scores between the two groups. Three days postoperatively, the AIPSI group had an average Harris score of 61.3 ± 8.7, while the Control group scored 62.4 ± 12.2 (*P* = 0.668). At 12 months postoperatively, the AIPSI group's average Harris score was 95.5 ± 3.1, compared to 96.0 ± 2.0 for the Control group (*P* = 0.431). No significant differences were observed in VAS pain scores from 3 days to 12 months postoperatively.

**Table 4:** Functional Outcome

	AIPSI group(*n* = 30)	Control Group(*n* = 30)	*P* Value
Pre-op Harris	56.2 ± 15.9	54.6 ± 13.4	0.668
Post-op Harris 3 d	61.3 ± 8.7	62.4 ± 12.2	0.672
Post-op Harris 1 m	80.4 ± 6.5	81.2 ± 7.5	0.647
Post-op Harris 3 m	89.9 ± 5.3	91.3 ± 3.6	0.247
Post-op Harris 12 m	95.5 ± 3.1	96.0 ± 2.0	0.431
Pre-op VAS	3.8 ± 1.6	4.1 ± 1.9	0.417
Post-op VAS 3 d	2.9 ± 1.4	2.6 ± 1.2	0.380
Post-op VAS 1 m	2.4 ± 1.1	2.5 ± 1.1	0.811
Post-op VAS 3 m	0.8 ± 0.9	0.8 ± 0.7	0.743
Post-op VAS 12 m	0.1 ± 0.3	0.3 ± 0.5	0.121

Presented as the mean ± standard deviations

## Discussion

Our findings demonstrate significant reductions in the number of bone cuts and reams in the PSI group, attributed to the precise localization of the femoral osteotomy position by the PSI guide, which leads to fewer repeat operations and potentially shorter surgery times. Currently, no significant differences in surgery duration and blood loss exist between the two groups. However, with increased surgical experience and improved operator proficiency, it is anticipated that surgery times may decrease. This study had some limitations; we utilized 3D preoperative planning based on CT data, whereas postoperative measurements were conducted using radiographs, which may affect the accuracy of the results.

This research represents the inaugural application of PSI in the DAA to THA. Uniquely, this study introduces metrics such as the number of bone cuts and reams, which are directly correlated with surgery duration, to assess the reliability of preoperative planning. According to Xi Chen et al. [12], surgery time and blood loss for PSI-assisted versus manual THA are similar; however, the PSI group may incur more bleeding due to the necessity for additional exposure and drilling.

For patients with DDH, especially those with high dislocation of the hips, placing the hip cup in a false acetabulum does not alleviate limb shortening and may lead to an increased likelihood of prosthesis loosening. Conversely, positioning the hip cup in the true acetabulum restores the anatomical center, equalizes limb lengths, and enhances the strength of the abductor muscles and walking pattern [13]. Although various researchers have developed methods to locate the true acetabulum, these techniques are complex, often necessitating larger PSI, specialized tools, and are generally restricted to patients with Crowe I type dysplasia [8, 14–16]. Our personalized navigation template, designed for use with smaller incisions in THA via a direct anterior approach, is compact and user-friendly; it requires only two guiding needles for fixation on the hip side, allows for the PSI template to be removed, and facilitates acetabular reaming in alignment with the screw guide's direction. Furthermore, we tailor the template to match the patient's specific skeletal structure, minimizing the influence of bone spurs to accurately pinpoint the cutting site and identify the true acetabulum's location for the surgeon. In cases of DDH patients with acetabular bone deficiencies, employing a graft block to reconstruct the acetabulum might be necessary.

In our study, which predominantly involved patients with Crowe I and II type dysplasia, we observed increased blood loss in the PSI group, with no significant differences in complications, pain, or functional activity levels. However, this feasibility study could be extended to encompass more complex cases, with the potential to enhance these outcomes. Lei Wang 's research [17] discovered that employing a 3D-printed titanium cup and liner for hip joint repair led to improved postoperative outcomes for patients, including higher Harris hip function scores, reduced pain as measured by the Visual Analog Scale (VAS), and better SF-36 quality of life scores, contributing to the restoration of hip joint function, pain alleviation, and enhanced patient quality of life. The presence of high dislocation and intricate acetabular morphology poses challenges for preoperative assessment and surgical planning. Utilizing artificial intelligence (AI) for preoperative planning, alongside the reconstruction of a complete pelvic model via 3D printing technology and personalized navigation templates, facilitates the development of a more detailed surgical plan.

Previous studies have indicated [9] that only 71% of femoral prostheses and 45% of acetabular prostheses accurately align with the preoperative plan when planning is done manually. In our study, the AIPSI group exhibited high precision in the planning and installation of prosthesis sizes. Allowing for a one-size discrepancy, the AIPSI group achieved an accuracy rate of 90% (27/30) compared to 60% (18/30) in the control group, with significantly greater precision in predicting the size of acetabular prostheses (Fig. 4E). The accuracy in predicting femoral stem prosthesis sizes was 93% (28/30) for the AIPSI group and 80% (24/30) for the control group, showing no significant difference (Fig. 4E). Previous research has demonstrated the successful establishment of a reliable hip joint rotation center and optimal acetabular cup positioning within an acceptable error margin using 3D printing assistance [18–20]. Accurate reconstruction of the acetabular position (anteversion and abduction angles) can diminish the risk of postoperative dislocation. Employing various techniques, such as Kirschner wires and lasers, for PSI placement guidance, the acetabular cup was placed within a 10° error margin in 90%-100% of cases in past studies [12, 21, 22]. Our study adopts a similar physical guiding technique to the Kirschner wire, but utilizes threaded fixation pins for enhanced stability, offering a considerable advantage in the accuracy of acetabular cup placement for the AIPSI group, with errors in inclination and anteversion angles below 5° in 25 instances. All of these cases fell within the safe zone (within 10°), in contrast to 23 cases in the control group.

The objective of performing THA encompasses not only alleviating pain and enhancing function but also accurately restoring the femoral offset (FO) and leg length. The incidence of patients with a leg length discrepancy greater than 10 mm was comparable between the two groups. However, the advantages of PSI assistance became more evident in the reconstruction of the FO. In conventional THA, 35–46% of patients surpassed the target range of ± 5 mm for FO [23, 24]. In our study, the precision of FO reconstruction exceeding ± 5 mm was 16.7% (5 / 30) in the AIPSI group compared to 57.7% (17/30) in the control group. A diminished FO can lead to decreased functionality and mobility, whereas an increase in femoral offset does not impact postoperative pain or function [25], although a significantly increased offset may hasten implant wear. Anisha B. Patel et al. [26] suggested that accurately restoring the femoral offset positively influences the range of motion (ROM), by mitigating impingement and enhancing abductor lever efficiency.

## Conclusion

Our research findings demonstrate that the use of PSI significantly aids surgeons in enhancing the precision of prosthetic implant placement, which helps in restoring the patient's leg length, and may offer additional insights and guidance for managing more complex anatomical variations or cases.

## Data Availability

All data generated or analysed during this study are included in this published article [and its supplementary information files].

## References

[1] Dandachli W, Kannan V, Richards R (2008). Analysis of cover of the femoral head in normal and dysplastic hips: new CT-based technique. J Bone Joint Surg Br.

[2] Grammatopoulos G, Alvand A, Monk AP (2016). Surgeons' Accuracy in Achieving Their Desired Acetabular Component Orientation. J Bone Joint Surg Am.

[3] Lanting BA, MacDonald SJ (2013). The painful total hip replacement: diagnosis and deliverance. Bone Joint J.

[4] Wang DD, Qian Z, Vukicevic M (2021). 3D Printing, Computational Modeling, and Artificial Intelligence for Structural Heart Disease. JACC Cardiovasc Imaging.

[5] Meng Meng, Jinzuo Wang, Tianze Sun, Wentao Zhang, Jing Zhang, Liming Shu, Zhonghai Li. Clinical applications and prospects of 3D printing guide templates in orthopaedics,Journal of Orthopaedic Translation, Volume 34,2022, Pages 22–41,ISSN 2214–031X, 10.1016/j.jot.2022.03.001. 10.1016/j.jot.2022.03.001 PMC9117878 35615638

[6] Lewinnek GE, Lewis JL, Tarr R (1978). Dislocations after total hip-replacement arthroplasties. J Bone Joint Surg Am.

[7] Meermans G, Konan S, Das R (2017). The direct anterior approach in total hip arthroplasty: a systematic review of the literature. Bone Joint J.

[8] Zhang YZ, Chen B, Lu S (2011). Preliminary application of computer-assisted patient-specific acetabular navigational template for total hip arthroplasty in adult single development dysplasia of the hip. Int J Med Robot.

[9] Wines AP, McNicol D (2006). Computed tomography measurement of the accuracy of component version in total hip arthroplasty. J Arthroplasty.

[10] Abdel MP, von Roth P, Jennings MT (2016). What Safe Zone? The Vast Majority of Dislocated THAs Are Within the Lewinnek Safe Zone for Acetabular Component Position. Clin Orthop Relat Res.

[11] Dastane M, Dorr LD, Tarwala R (2011). Hip Offset in Total Hip Arthroplasty: Quantitative Measurement with Navigation. Clin Orthop Relat Res.

[12] Chen X,Li S,Wang Y, et al. Artificially Intelligent Three-Dimensionally -Printed Patient-Specific Instrument Improves Total Hip Arthroplasty Accuracy. J Arthroplasty. 2022. 10.1016/j.arth.2022.12.017. 10.1016/j.arth.2022.12.017 36535443

[13] Stans AA, Pagnano MW, Shaughnessy WJ (1998). Results of total hip arthroplasty for Crowe Type III developmental hip dysplasia. Clin Orthop Relat Res.

[14] Kunz M, Rudan JF, Xenoyannis GL (2010). Computer-assisted hip resurfacing using individualized drill templates. J Arthroplasty.

[15] Kunz M, Balaketheeswaran S, Ellis RE (2015). The influence of osteophyte depiction in CT for patient-specific guided hip resurfacing procedures. Int J Comput Assist Radiol Surg.

[16] Small T, Krebs V, Molloy R (2014). Comparison of acetabular shell position using patient specific instruments vs. standard surgical instruments: a randomized clinical trial. J Arthroplasty..

[17] Wan L, Wu G, Cao P (2019). Curative effect and prognosis of 3D printing titanium alloy trabecular cup and pad in revision of acetabular defect of hip joint. Exp Ther Med.

[18] Zampelis V, Flivik G (2021). Custom-made 3D-printed cup-cage implants for complex acetabular revisions: evaluation of pre-planned versus achieved positioning and 1-year migration data in 10 patients. Acta Orthop.

[19] Durand-Hill M, Henckel J, Di Laura A (2020). Can custom 3D printed implants successfully reconstruct massive acetabular defects? A 3D-CT assessment. J Orthop Res.

[20] Li Q, Chen X, Lin B (2019). Three-dimensional technology assisted trabecular metal cup and augments positioning in revision total hip arthroplasty with complex acetabular defects. J Orthop Surg Res.

[21] Spencer-Gardner L, Pierrepont J, Topham M (2016). Patient-specific instrumentation improves the accuracy of acetabular component placement in total hip arthroplasty. Bone Joint J.

[22] Hananouchi T, Saito M, Koyama T (2010). Tailor-made Surgical Guide Reduces Incidence of Outliers of Cup Placement. Clin Orthop Relat Res.

[23] Cassidy KA, Noticewala MS, Macaulay W (2012). Effect of femoral offset on pain and function after total hip arthroplasty. J Arthroplasty.

[24] Liebs TR, Nasser L, Herzberg W (2014). The influence of femoral offset on health-related quality of life after total hip replacement. Bone Joint J.

[25] Martin JR, Camp CL, Wyles CC (2016). Increased Femoral Head Offset is Associated With Elevated Metal Ions in Asymptomatic Patients With Metal-on-Polyethylene Total Hip Arthroplasty. J Arthroplasty.

[26] Patel AB, Wagle RR, Usrey MM (2010). Guidelines for implant placement to minimize impingement during activities of daily living after total hip arthroplasty. J Arthroplasty.

